# Comparative assessments of indel annotations in healthy and cancer genomes with next-generation sequencing data

**DOI:** 10.1186/s12920-020-00818-6

**Published:** 2020-11-10

**Authors:** Jing Chen, Jun-tao Guo

**Affiliations:** Department of Bioinformatics and Genomics, University of North Carolina at Charlotte, 9201 University City Blvd, Charlotte, NC 28223 USA

**Keywords:** Indel, Insertion, Deletion, Germline variants, Somatic variants, Cancer

## Abstract

**Background:**

Insertion and deletion (indel) is one of the major variation types in human genomes. Accurate annotation of indels is of paramount importance in genetic variation analysis and investigation of their roles in human diseases. Previous studies revealed a high number of false positives from existing indel calling methods, which limits downstream analyses of the effects of indels on both healthy and disease genomes. In this study, we evaluated seven commonly used general indel calling programs for germline indels and four somatic indel calling programs through comparative analysis to investigate their common features and differences and to explore ways to improve indel annotation accuracy.

**Methods:**

In our comparative analysis, we adopted a more stringent evaluation approach by considering both the indel positions and the indel types (insertion or deletion sequences) between the samples and the reference set. In addition, we applied an efficient way to use a benchmark for improved performance comparisons for the general indel calling programs

**Results:**

We found that germline indels in healthy genomes derived by combining several indel calling tools could help remove a large number of false positive indels from individual programs without compromising the number of true positives. The performance comparisons of somatic indel calling programs are more complicated due to the lack of a reliable and comprehensive benchmark. Nevertheless our results revealed large variations among the programs and among cancer types.

**Conclusions:**

While more accurate indel calling programs are needed, we found that the performance for germline indel annotations can be improved by combining the results from several programs. In addition, well-designed benchmarks for both germline and somatic indels are key in program development and evaluations.

## Background

Insertion and deletion (indel) is the second-largest genetic variation type in human genomes. On average, one healthy human genome differs from the reference genome at about 566,000 sites with indel lengths ranging from 1 to 1000 base pairs (bps) [1]. Typically, small indels are termed for insertions/deletions of shorter than 50 bps while longer ones are considered as structural variants (SVs) [2, 3]. Besides contributing to genetic variations in healthy population, deleterious indels in both coding and non-coding regions can lead to various types of diseases. For example, coding indels were identified in breast cancer development genes, including AKT1, BRCA1 and CDH1, and the fragile X syndrome is caused by a large insertion in 5′UTR of the FMR1 gene [4, 5]. Several databases with annotated indels have been developed to document these variants, including dbSNP, dbVar, and COSMIC (the Catalogue Of Somatic Mutation In Cancer) [6–8].

Detection of genomic variations including indels represents one of the most important aspects in human genome analysis. Mills et al*.* reported 2 million unique indels in their updated analysis of 79 genomes in 2011 [9, 10]. The indel set from these 79 genomes is commonly used as a reference for indel analysis since these indels were annotated with Sanger sequencing data, which reported a 97.2% validation rate [10]. There are also studies focused on somatic indels in cancer genomes. For example, Niu et al*.* analyzed 4201 non-frame-shift indels and identified more than 6000 mutation clusters on protein 3-dimentional (3D) structures across 19 cancer types [11]. Besides somatic coding indels, non-coding indels also play important roles in cancer genomes. Imielinski et al*.* found that non-coding somatic indels tend to be enriched in lineage-defining genes in multiple cancer genomes [12].

Next-generation sequencing (NGS) technology has reduced the sequencing cost and produced more genome sequence data. A number of programs have been developed for both germline indel and somatic indel identification from NGS data [25–27]. Current indel calling programs use different algorithms to distinguish sequence errors or alignment errors from real indel variations [28]. General indel calling programs are classified into five major groups: alignment-based methods, split read mapping methods, paired end mapping methods, haplotype based methods, and machine learning-based approaches [25, 28]. A list of indel calling programs with variant types that can be detected and the corresponding algorithms are shown in Table 1 [13, 15–20, 22–24]. Alignment-based methods, including Dindel, GATK_UG, SAMTools and Varscan, use information from the mapping step and identify indels with statistical models [25]. These alignment-based programs differ in the statistical models and processing details [18]. The indel sizes from these alignment-based programs are constrained by the length of sequence reads. Consequently the medium sized indels and large insertions are hard to detect since the workflow relies on the initial alignments [28]. Split read mapping methods, such as Pindel, rely on the discordant reads in the alignment step and can be used to annotate medium sized indels. These methods usually do not use statistical approaches to filter variants [16]. The haplotype-based methods, such as GATK_HC and Platypus, collect candidate haplotypes and identify the variants based on the realignment results on haplotypes [25]. Paired-end read mapping methods compare the real and expected distances between paired-end reads to identify potential indels. However the exact indel sequences are usually hard to annotate. They are considered more accurate for medium sized indels but not for small indels. Machine learning methods need training data to predict true indels [25, 28]. Due to these issues or constraints, paired-end read mapping and machine learning-based methods are not included in this study.

**Table 1:** A list of indel calling programs

Programs	General /somatic	Type of variants	Core algorithms	Notes and references
Dindel	General	Indel	Alignment-based	Bayesian approach [13]
GATK_HC	General	SNP + Indel	Haplotype-based	Collection of candidate haplotypes [14]
GATK_UG	General	SNP + Indel	Alignment-based	Bayesian genotype likelihood model [15]
Pindel	General	Indel	Split read mapping	A pattern growth approach [16]
Platypus	General	SNP + Indel	Haplotype-based	Collection of candidate haplotypes [17]
SAMTools	General	SNP + Indel	Alignment-based	Bayesian model [18]
Varscan	General	SNP + Indel	Alignment-based	Heuristic method [19]
GATK Mutect2	Somatic	SNP + Indel	Allele frequency	Re-assembly of haplotypes methods [20, 21]
Strelka	Somatic	SNP + Indel	Allele frequency	Bayesian approach [22]
Strelka2	Somatic	SNP + Indel	Allele frequency	A mixture model [23]
Varscan2	Somatic	SNP + Indel	Heuristic methods	Heuristic and statistical methods [24]

Besides general indel calling programs, there are tools designed for detecting germline/somatic variants from cancer genomes. Almost all somatic indel calling programs can detect single nucleotide variants, some of them can also detect SVs [29]. Majority of these programs use tumor-normal paired sample data to identify somatic variants, while others can predict with only tumor samples [30]. For programs based on the tumor-normal paired data, the general core algorithms include joint genotype analysis, allele frequency analysis, heuristic threshold, haplotype analysis, and machine learning [30]. In this study, we selected Varscan2, GATK Mutect2, Strelka and Strelka2 for comparative somatic indel analysis based on their good performances reported by several groups [21, 29, 31–34] (Table 1). In general, performance evaluations for somatic indel identification can be done with simulation data and/or real sequence data [31, 33]. While the simulation data can help test different features such as variant allele fractions [33], comparison of indel annotation methods with real NGS data can provide useful guidance for their application in variant analysis in disease genomes. Even though currently there is no gold standard for evaluating somatic indel variants from cancer genomes, several existing databases can provide some useful information [31]. For instance, the annotated indels in GATK Resource Bundle and dbSNP can be used to check false positive cases and indels in COSMIC can be used to evaluate positive cases, respectively [7, 8, 31, 35]. However, caution should be taken when using these databases for evaluation purpose as both databases contain only partial data.

**Fig. 1:**
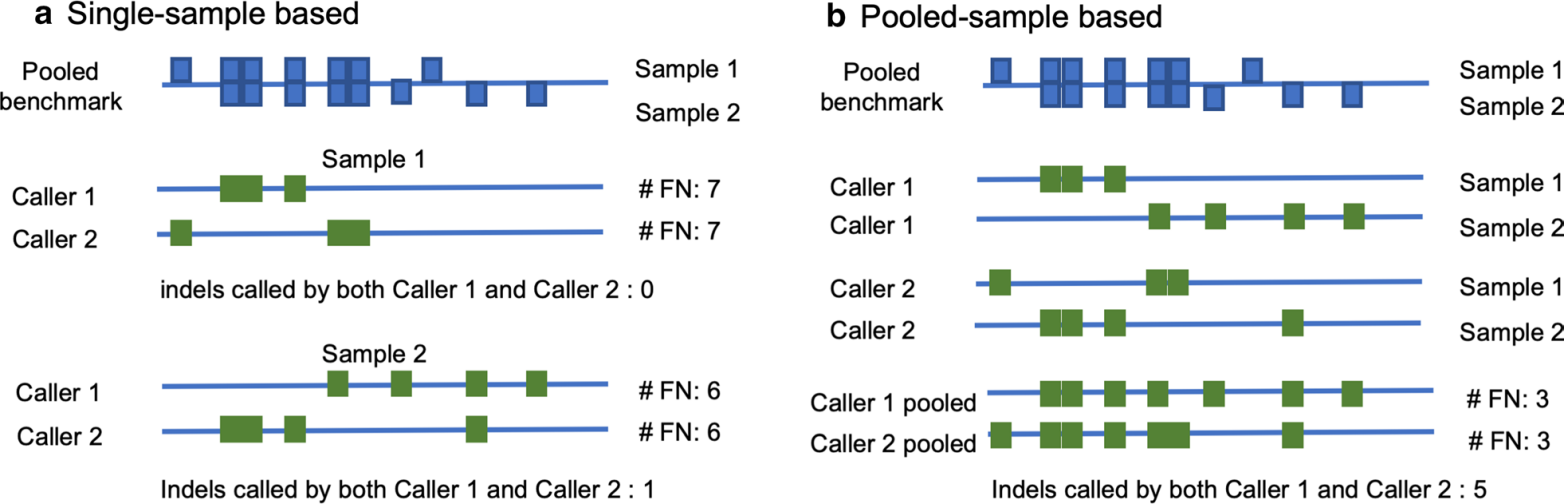
Comparison of different methods regarding false negative indels. A schematic comparison between single-sample based method (**a**) and pooled-sample based method (**b**) with a pooled reference benchmark. FN: false negative

Accurate annotation of indels is of paramount importance in studying genetic variations and in identifying disease associated indels [36–38]. To test the consistency or differences among the general indel calling programs, Hasan et al*.* performed a comparative analysis by using the sequences of chromosome 11 from 78 samples of the 1000 Genomes Project and showed that 78–89% of the benchmark indels are not identified in a sample by any program and only a very small number of indels are identified by all seven programs [25]. However, the results do not accurately reflect the performance of each program as well as the common indels predicted from different programs. First, they compared the indels from individual genome samples to the pooled indel dataset of 79 genomes. Rare and low frequency variants account for a large proportion of indels and the pooled indel set includes all of them, but an individual sample may contain only a small subset of the pooled indel set [1, 39, 40]. Figure 1a shows a schematic example to explain the potential pitfalls of comparing individual samples with a pooled reference set from multiple samples. In this study we applied a pooled-sample based method for more accurate comparative analysis since indels from multiple samples from one program are pooled together to compare with the pooled benchmark indels (Fig. 1b). In addition, we expanded the comparison with the whole genome sequences instead of only one chromosome.

Unlike SNPs, indels are more complicated in that there are two different indel types, insertion and deletion. Moreover, for a coding indel, it can be a frame-shift (FS) or non-frame-shift (NFS) indel. Consequently, the way to compare the indels can affect the number of true positives and false positives. Previous studies used a position range of *i* ± 5 (where *i* is the indel position) to determine if an indel is the same one as that in the reference set [41]. However, this approach has several disadvantages. First, the indel types, insertion or deletion, are not considered separately. An insertion and a deletion at the same genome position are two different indels, not the same indel. Secondly, for coding indels, 1 bp difference in a position may result in a totally different protein sequence due to an open reading frame shift. In light of these issues, we adopted a modified approach by considering indel types (insertion or deletion) as well as positions, which is especially important in germline indel analysis.

## Methods

### Datasets

We used the same dataset as Hasan et al., which consists of 78 samples from the 1000 Genomes Project (https://www.internationalgenome.org/) covering five super populations (EUR, EAS, SAS, AMR, and ARF) and 26 sub-populations (three from each sub-populations) to evaluate general indel calling programs [25]. The benchmark is a set of small indels identified by Mills et al. [10]. For somatic indel program evaluation, we used a total of 30 tumor-normal paired data, including 10 colon cancer, 10 breast cancer, and 10 bladder cancer samples. The cancer genome sequencing data were downloaded from TCGA with dbGap ID phs000178.v11.p8. A total of 4970 indels from the latest version of COSMIC (v90) were downloaded for somatic indel evaluations [8].

### Evaluation methods

For germline indels from healthy genomes, they are mainly genetic variants with the type and position of the indels presumably conserved in sub-populations or super populations. In other words, they are less random compared with somatic variants and usually do not lead to diseases. Therefore, when evaluating germline indels from healthy genomes, we only counted the indels that are located at the same positions with the same insertion or deletion sequences between the samples and the reference as positive identifications. Since somatic indels from cancer genomes are less conserved than the germline indels, we used the typical range of *i* ± 5 in positions along with the indel types, either insertion or deletion, for comparative evaluation.

Recall, precision and F measure are calculated for performance evaluations (Eqs. 1–3):

1 $$Recall=\frac{TP}{TP+FN}$$

2 $$Precision=\frac{TP}{TP+FP}$$

3 $$F=\frac{2\times Recall \times Precision}{Recall+Precision}$$

where TP represents true positive, FP represents false positive, and FN represents false negative. As mentioned in the Background section, for germline indels, the TP, FP and FN are identified by a pooled sample-based method (Fig. 1b). For somatic indel evaluation, the predicted indels are compared with the annotated indels in the COSMIC database as potential somatic indels (the indel types are classified using the indel labels downloaded from COSMIC). To identify potential false somatic indels, we compared the predictions with the indel set from the GATK Resource Bundle, which is considered as a standard germline indel set for human reference GRCh38 [35].

## Results

### General indel calling programs

#### Overall analysis of the predicted indels

The number of true positive and false positive indels from healthy genomes by different programs is listed in Table 2. SAMTools calls the largest number of indels, with Platypus ranks the second. The number of the TP indels varies by programs. Dindel has the highest recall (0.78) but with a low precision (0.24). Varscan, which calls the least number of indels, has the highest precision (0.56) as well as the best F value (0.48). GATK_UG and GATK_HC have the second-best F value with relatively good recall and precision .

**Table 2:** Performance of different general indel annotation programs

Tool	TP indels	FP indels	Recall	Precision	F
Varscan	533,101	**424,740**	0.42	**0.56**	**0.48**
GATK_UG	884,763	1,802,477	0.69	0.33	0.45
GATK_HC	948,738	2,026,903	0.74	0.32	0.45
Pindel	446,622	619,846	0.35	0.42	0.38
Dindel	**994,947**	3,097,117	**0.78**	0.24	0.37
Platypus	941,046	3,403,565	0.74	0.22	0.33
SAMTools	930,860	15,083,658	0.73	0.06	0.11

The bold represents the highest value in each column

**Fig. 2:**
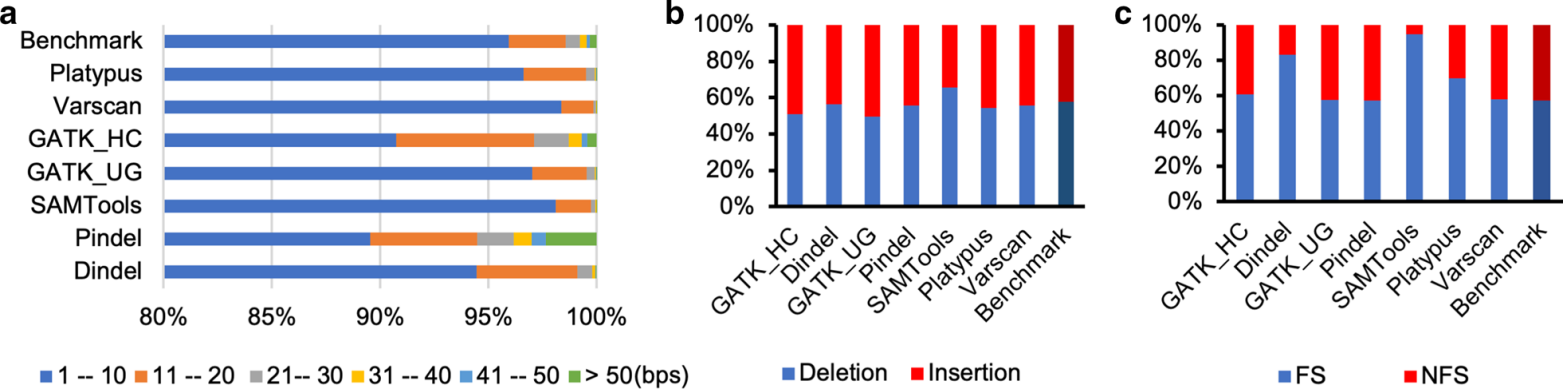
Comparisons of indels from seven general indel calling programs. **a** Indel size distribution. **b** Indel type distribution. **c** Coding indel type distribution. *FS* frame shift, *NFS* non-frame shift

Among all the programs, GATK_HC calls the longest indel with 616 bps. The length distribution is shown in Fig. 2a (percentages) and Additional file 1: Table S1 (counts) with the benchmark as a reference. SAMTools has the largest number of short indels for length between 1 and 20 bps, which is not surprising since it calls much more indels than any other programs (Table 2 and Additional file 1: Table S1). Pindel predicts the largest number of indels longer than 50 bps, largely because Pindel uses an algorithm that tends to call longer indels. In terms of mid-length indels between 20 and 50 bps, GATK_HC has the largest number in each category. Percentage-wise, Platypus, Varscan, GATK_UG and SAMTools predict relatively more short indels compared to other three programs. We also compared the programs in terms of indel types, insertion and deletion (Fig. 2b and Additional file 1: Table S2). SAMTools has a higher percentage of deletion types while GATK_UG has more insertion types in terms of the ratio when compared with the benchmark. Dindel has the most similar insertion/deletion ratio (56.2%/43.8%) to the benchmark (57.6%/42.4%) and it has the highest TP rate for both insertion and deletion types (Additional file 1: Table S2).

In coding regions, indels can be grouped into FS and NFS types. An NFS indel consists of a multiple of three base pairs, introducing an insertion or deletion of one or more amino acids while keeping the other part of the protein sequence unchanged. In contrast, an FS indel changes the reading frame starting from the site of insertion/deletion, which can produce different protein sequences from the indel position. FS indels can also lead to premature termination and the mRNA molecules can be subjected to a surveillance pathway called non-sense-mediated mRNA decay (NMD) [42]. The proportion of NFS and FS coding indels from each program is shown in Fig. 2c and Additional file 1: Table S3. GATK_UG, Pindel and Varscan show similar FS/NFS ratios to that of the benchmark while Pindel, SAMTools, and Platypus have a much higher percentage of FS coding indels.

#### Pare-wise comparisons

To check the similarity or difference of indels predicted by two different programs, the overlapped indels from two programs are compared with the benchmark indels. The recall and precision values are presented in Table 3, showing a trade-off between recall and precision. When a program is paired with Varscan or Pindel, it usually achieves high precision with smaller number of FPs while having low recall at the same time since these are the two programs that call the lowest number of total indels. The indels from Varscan, GATK_UG and Dindel are highly similar. About 94% of indels from Varscan are also annotated by GATK_UG (898,482 out of 957,841) or Dindel (903,756 out of 957,841).

**Table 3:** Pair-wise comparison between general indel calling programs

Recall	Varscan	GATK_UG	GATK_HC	Pindel	Dindel	Platypus	SAMTools
*Precision*							
Varscan	–	0.40	0.41	0.30	0.41	0.39	0.36
GATK_UG	0.57	-	0.65	0.33	0.66	0.64	0.60
GATK_HC	0.59	0.43	–	0.34	0.72	0.68	0.65
Pindel	0.62	0.59	0.57	–	0.34	0.33	0.31
Dindel	0.58	0.41	0.38	0.56	–	0.70	0.68
Platypus	0.57	0.34	0.38	0.59	0.35	–	0.66
SAMTools	0.55	0.40	0.40	0.60	0.27	0.29	–

#### Combination of indels from different programs

**Fig. 3:**
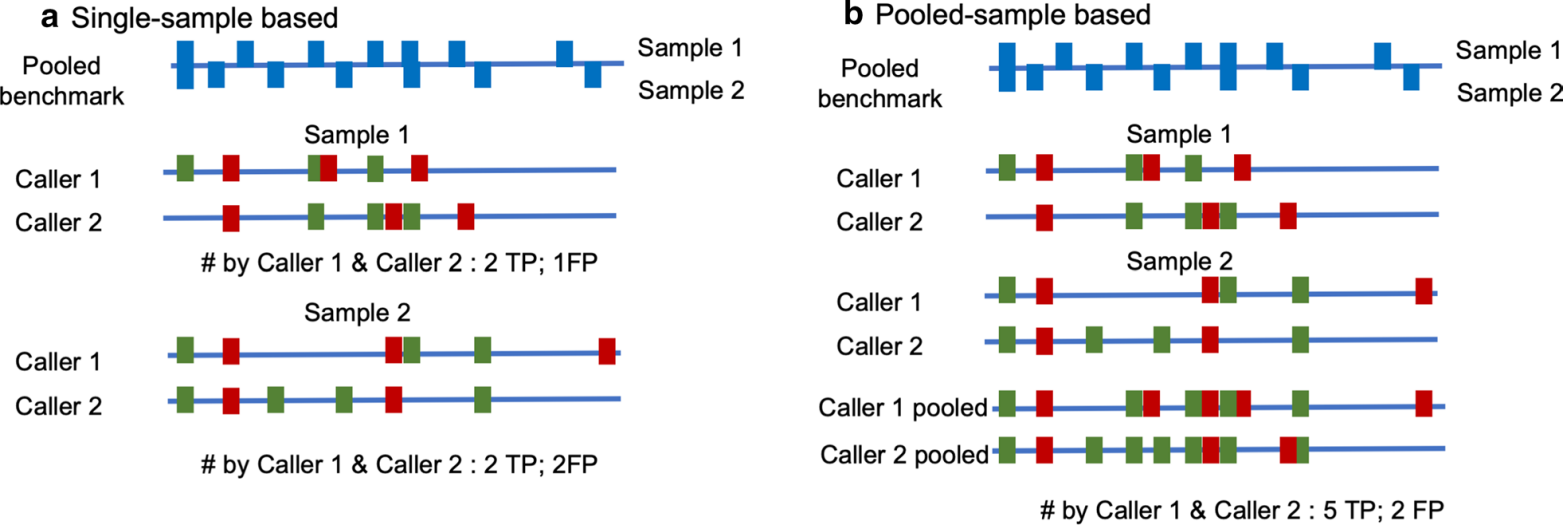
Comparison of different methods for common indels from different programs. A schematic comparison between single-sample based method (**a**) and pooled-sample based method (**b**) with a pooled reference benchmark. Green represents true positives. Red represents false positive predictions. Blue blocks are the benchmark indels

The results from individual programs have shown that there are a large number of false positive indel predictions from the NGS data (Table 2). While false negatives may represent missed opportunities, false positives can result in wrong conclusions and are costly in real applications. We hypothesize that by selecting the consistent indel annotations from different programs, we may be able to remove majority of the false positives while retaining most of the true positives. The underlying idea is that in general, unlike false positives, true indels can be identified by different prediction algorithms. The ones that are program specific have a higher probability to be false positives. In a previous study, Hasan et al*.* showed that only a very small number of indels were called by all seven programs [25]. But as discussed in Background, that conclusion is a result from their approach by comparing the indels from individual samples to the pooled benchmark dataset, which may produce a large number of false negatives. We adopted a pooled sample method for a more meaningful comparison in this study. Figure 3 shows a schematic example to explain the differences by counting the overlaps or consistent indels between the two approaches. Among the seven indels called by both caller 1 and caller 2 with the pooled sample method, five of them are true positive indels. However, the single sample approach only identifies two true positives, resulting a very low TP rate from the overlapped indels (Fig. 3).

Table 4 shows the averages of TP indels, FP indels, recall, precision and F values for all possible combinations including individual programs. The results from Hasan et al. show that only a small proportion of TP indels (1.51%) are called by all seven programs [25]. With our pooled sample approach, we found that 476,253 indels are called by all seven programs. Among these indels, 326,184 can be found in the reference set, representing a TP rate of 25.6%.

**Table 4:** Performance comparison of different program combinations (showing average values)

# of Tools	TP indels	FP indels	Recall	Precision	F
1	811,440	3,779,758	0.64	0.31	0.37
2	639,772	899,660	0.51	0.48	0.45
3	528,467	496,588	0.41	0.56	0.45
4	450,280	322,289	0.37	0.60	0.44
5	394,064	230,561	0.31	0.64	0.41
6	354,111	179,699	0.28	0.67	0.38
7	326,184	150,069	0.26	0.68	0.37

**Fig. 4:**
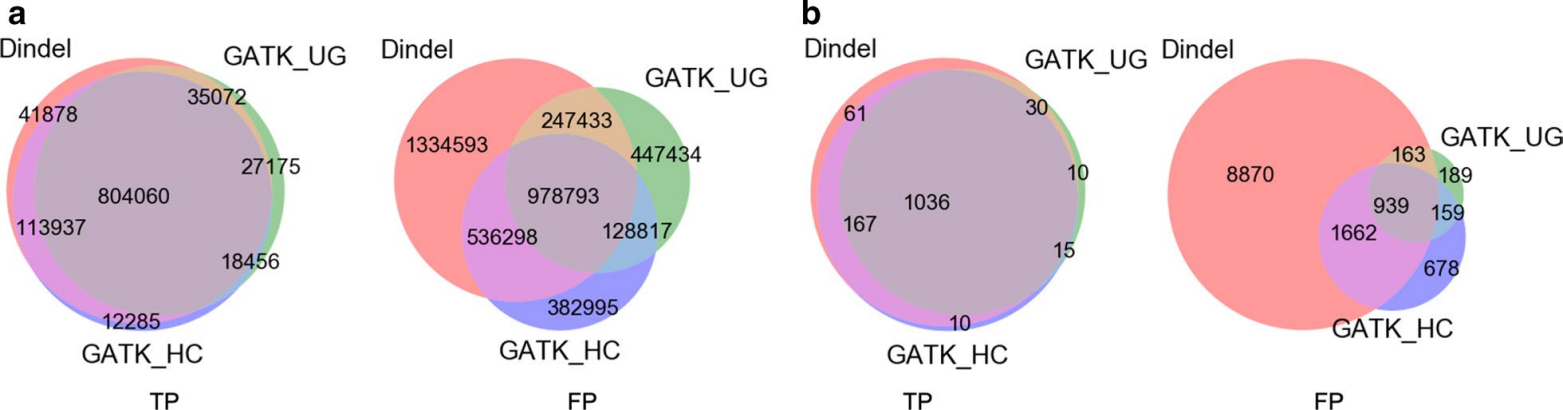
Overlapped indels by GATK_UG, GATK_HC and Dindel. **a** All indels; **b** coding indels only

Among all the possible combinations, including the individual programs, a five tool combination of GATK_UG, GATK_HC, Pindel, SAMTools and Dindel has the highest F value (0.53). Dindel has the highest recall (0.78, Table 2) and a combination of three tools (GATK UG, Pindel and SAMTools) has the highest precision (0.69). On average, a combination of 2 or 3 programs has the highest average F values (Table 4). Table 5 lists top three combinations of two and three programs ranked by F values. As shown in Tables 4 and 5, adding more programs can remove more false positives than true positives and a combination of three programs seems to have a good balance of recall and precision. Figure 4 shows an example of indels called by 3 programs: GATK_UG, GATK_HC and Dindel. There are large overlaps among the TP indels either for all indels (Fig. 4a) or for coding indels only (Fig. 4b), while the disagreement among the FP indels are much bigger. Therefore, if a low number of false positives is preferred in an application, results from more programs can be used and combined.

**Table 5:** Top 3 indel annotation program combinations (2 programs and 3 programs)

F rank	Combination of 2 tools	TP	FP	Recall	Precision	F
1	GATK_UG + GATK_HC	822,516	1,107,610	0.65	0.43	0.51
2	GATK_UG + Dindel	839,132	1,226,226	0.66	0.41	0.50
3	GATK_HC + Platypus	871,596	1,403,334	0.68	0.38	0.49

F rank	Combination of 3 tools	TP	FP	Recall	Precision	F
1	GATK_UG + GATK_HC + Dindel	804,060	978,793	0.63	0.45	0.53
2	GATK_UG + GATK_HC + SAMTools	725,419	768,246	0.57	0.49	0.52
3	GATK_UG + GATK_HC + Platypus	778,439	991,540	0.61	0.44	0.51

### Somatic indel calling programs

Unlike general indel calling approaches, the majority of somatic indel annotations need both normal and diseases genome samples and thus are more complicated. Different methods or algorithms have been developed for somatic indel identifications (Table 1). In this study, we applied four somatic indel calling programs to three types of cancers. As discussed in Background, there are no benchmark sets available to assess the true positive or false positives for cancer somatic genome indels. But for comparison purposes between programs and cancer types, we can use the COSMIC database with annotated somatic cancer indels and GATK Resource Bundle as potential false positives (or germline indels) to see how much they agree or differ with each other. Since the COSMIC indel set represents only a small portion of real cancer population indels, a small number of indels in COSMIC does not necessarily indicate a large number of false positives from a program. Similarly, an indel found in the germline indel set does not necessarily mean it is a true false positive since there is a single cancer sample vs. pooled germline samples problem. Nevertheless, the comparative analysis can provide some insights about these somatic indel calling programs and the similarity or differences among different cancer types.

The number of potential true positive and false positive indels called by four programs are shown in Table 6. GATK Mutect2 calls the largest number of indels independent of cancer types and it has the largest overlap with the COSMIC indels and relatively low number of potential germline indels among the four programs. Strelka2 has the smallest numbers of indels for bladder and breast cancer types while Varscan2 calls the lowest number of indels in colon cancer. In terms of cancer types, colon cancer has more indels than the other two cancer types. The number of indels in bladder cancer is much less than the other two types. Taken together, GATK Mutect2 has a better coverage of somatic indels in all three cancer types with relatively low number of germline indels, or potential false positives. Strelka has the second largest number of total indels and COSMIC indels, however, the number of potential germline indels is also high.

**Table 6:** Performance comparison of different somatic indel annotation programs

Tools	Total indels	Cancer type	COSMIC indels	Potential germline indels and rate
Strelka	2,186	Bladder	5	884 (0.40)
	5,521	Breast	5	2536 (0.46)
	14,174	Colon	11	5227 (0.37)
Strelka2	867	Bladder	0	225 (0.26)
	2162	Breast	0	768 (0.36)
	9920	Colon	2	3583 (0.36)
Varscan2	1804	Bladder	2	438 (0.24)
	3796	Breast	4	879 (0.23)
	6286	Colon	8	831 (0.13)
Mutect2	19,124	Bladder	10	761 (0.04)
	44,373	Breast	16	1708 (0.04)
	30,503	Colon	31	4971 (0.16)

**Fig. 5:**
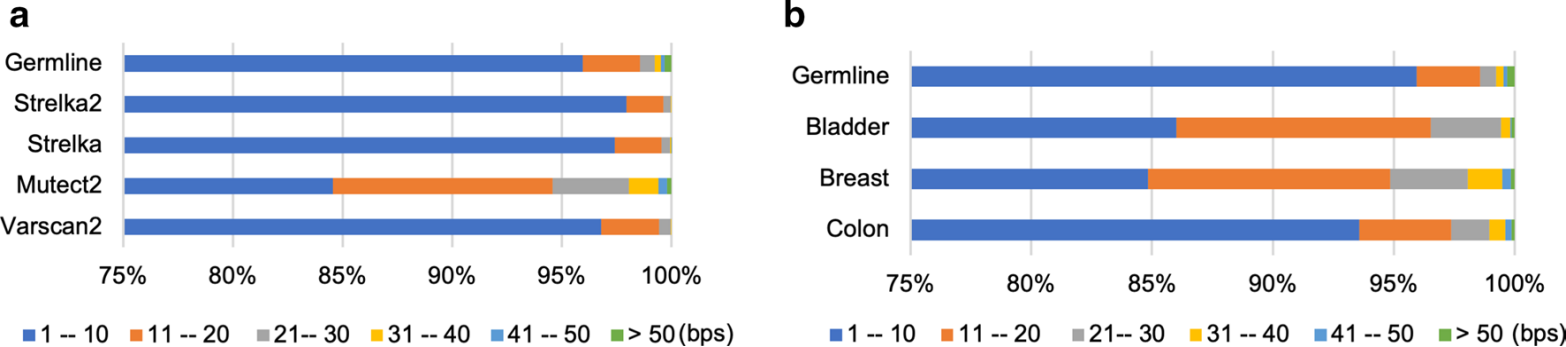
Somatic indel size distribution. **a** Program based; and **b** cancer type based

As for the length distribution of the somatic indels, GATK Mutect2 calls the longest somatic indel (245 bps) in a cancer genome and identifies more longer indels (Fig. 5a and Additional file 1: Table S4). It has 202 indels longer than 50 bps. However, no other programs identify any indels of length 50 or more. The length distributions in terms of cancer types also vary. Even though colon cancer has the largest number of indels, breast cancer has more longer indels (Fig. 5b and Additional file 1: Table S4).

In healthy genomes, there are more germline deletions (57.75%) than germline insertions (42.25%) (Additional file 1: Table S5) while in cancer indel database COSMIC, the ratios are slightly different with 34.39% of insertions and 52.00% of deletions, with the remaining cases assigned as complex indels (13.61%) (Additional file 1: Table S5) [39]. Except for GATK Mutect2 in bladder and breast cancer genomes, all other programs detect relatively low number of insertions. It is not clear if cancer genomes have relatively fewer insertions or the programs have difficulty in identifying somatic insertions. As for coding indels, germline coding indels has slightly more NFS indels (51.63%) than the FS indels (48.37%) (Additional file 1: Table S6). It is not surprising that the number of short FS coding indels is smaller than expected (2 to 1 ratio if there is no selection) in healthy genomes, as FS indels are more deleterious than NFS indels, which are more likely to be removed from the population during evolution. FS indels found in healthy individuals generally are less deleterious and contribute to phenotypic diversity through different ways [39]. In COSMIC cancer indel database, FS indel is the dominant coding indel type (81.05%). Except for Strelka2 in balder cancer, all other programs predict more FS indels than NFS indels in all three cancer types. It should be pointed out that the total numbers of coding indels predicted by Varscan2 and Strelka2 are rather small (Additional file 1: Table S6).

**Fig. 6:**
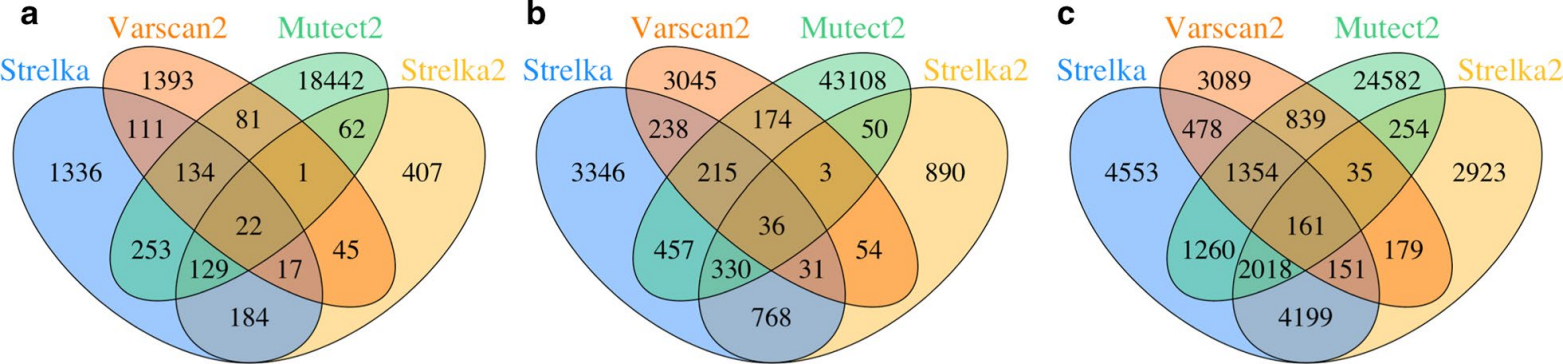
Overlapped indel annotations of different cancer types. **a** Bladder cancer; **b** breast cancer; and **c** colon cancer

When the somatic indels from different programs are compared, the number of similar indels from different programs or the overlapped indels are much smaller especially when more programs are considered (Fig. 6 and Table 7). This is quite different from the germline indels by the general indel annotation programs especially the comparison criteria are not as stringent as those used for germline indel comparisons (Table 4), in which there are a large number of indels called by all the programs, especially for the true positive indels. Table 7 and Fig. 6 show that when all four programs are used, there are only 22, 36, 161 indels in the bladder, breast, and colon cancel samples respectively. These results suggest that the agreement among different programs is low and it might not be practical to use multiple programs in order to remove false positives in cancer samples as we showed in the germline indel cases since it also dramatically decrease the total number of indels as well as true positives.

**Table 7:** Performance on different number of somatic program combinations (The data shown are average values)

Cancer types	# of Tools	Total indels	COSMIC indels	Potential germline indels and rate
Bladder	1	5995	4	577 (0.24)
	2	285	1	64 (0.22)
	3	92	1	20 (0.24)
	4	22	0	6 (0.27)
Breast	1	13,963	6	1463 (0.27)
	2	616	1	185 (0.25)
	3	181	1	47 (0.19)
	4	36	0	5 (0.14)
Colon	1	15,221	13	6666 (0.26)
	2	3142	3	948 (0.23)
	3	1051	2	300 (0.18)
	4	161	1	14 (0.09)

## Discussion

Accurate annotation of indels in both healthy and cancer genomes is important for downstream analysis in biological and medical applications. A number of programs have been developed for identifying indels from both healthy genomes for germline indels as well as cancer genomes for somatic indels with NGS data. Comparative analysis and evaluation can provide useful information about each program’s performance. The best available benchmark for large-scale germline indels so far is the pooled sample indels [10]. One previous comparative study applied this pooled benchmark set and evaluated seven general indel calling programs using chromosome 11 of 78 samples. However, the comparison was carried out between a single sample and the pooled benchmark, which is problematic as shown in Figs. 1 and 3. It may also explain why the study finds little overlap when the indels from all seven tools are combined [25]. In this study we carried out an improved approach to assess the general indel calling programs using the whole genome NGS sequences instead of using one chromosome sequences. More importantly, we adopted a pooled sample vs. pooled benchmark comparison, which provides more accurate assessment of programs’ performances. The new method greatly reduced the number of false negative cases by correctly recognizing the true positives (Figs. 1, 3). Last but not the least, we adopted a stringent indel comparison approach by considering the exact indel position as well as the indel types, which was not considered in previous studies. It should be noted that even though we applied a pooled sample approach, the comparison is not error free since the samples and the genomes in the benchmark set are different. There are some sample specific indels in both the test set and the benchmark set. Nevertheless, our approach makes the best use of the reference set and provides more accurate performance evaluations.

These general indel calling programs employ different prediction algorithms and predict different number of indels with different length and type distributions (Additional file 1: Tables S1-2). There is a tradeoff between the number of true positives and false positives. Some of them recognize a large number of true positive indels but at the same time output more false positive indels. We found that combing indels predicted from several different programs can achieve a good balance of TPs and FPs by removing a large number of false positives while keeping most of the true positives. The idea behind this is that if an indel is a true one, most programs are expected to find it no matter what algorithm is used. On the other hand, if an indel is a false one, it probably will only be predicted by one or a small number of programs. Our results show it is indeed the case and the best TP/FP balance is achieved with two or three different programs (Tables 4, 5).

In addition to germline indels, we also carried out program comparisons of somatic indel predictions using 30 cancer samples of three different types. Evaluating somatic indels is even more challenging because there is no benchmark that can be used for a systematic comparison and cancer indels are more random in terms of indel positions. Nevertheless, by using a common sample sets, we can evaluate the similarity/differences of indels from different somatic indel calling programs and among different cancer types. To get a sense of the potential number of true positive or false positive somatic indels, we compared the predicted indels with the cancer indels in COSMIC database (as potential true positives) and the germline indel set (as potential false positive somatic indels). While each program produces different number of indels with various ratios of indel types (Table 6 and Additional file 1: Tables S4 and S5), there is a clear trend among different cancer types in general. Bladder cancer has the lowest number of predicted somatic indels and colon cancer has the largest number of predicted somatic indels (Tables 6 and Additional file 1: Table S5). Secondly, unlike the germline indels, the number of indels predicted by all programs is very small (Table 7), suggesting a low agreement among the programs even though the input sequences are the same. Thirdly, the programs identify a small number of insertions. This trend has also been reported by other case studies. For example, 2233 deletions and 544 insertions were identified from 21 breast cancer genomes by a modified Pindel program, and 680 deletions and 303 insertions were found from a skin cancer genome by Pindel, BWA and GROUPER [16, 43–45]. In COSMIC database, there are also less insertions compared with deletions (Additional file 1: Table S5). On the other hand, Sathya et al. identified SNP and indel patterns from lung cancer genomes and found more insertions than deletions in both healthy genomes and lung cancer genomes using GATK-UG [46]. Whether the difference in the ratio of insertion and deletion in the cancer genomes is caused by the characteristics of the cancer genomes or by the algorithms used by the somatic variants calling programs remains to be further studied.

## Conclusions

Our results show that a better balance between TP and FP can be achieved by combining results from a small number of programs for germline indel annotations. However, the low agreement among indel calling programs, especially for somatic indel identifications, calls for novel approaches for improving prediction accuracy with NGS data. In addition, the development of such approaches needs well-annotated indel reference sets.

## Supplementary information

**Additional file 1.** Supplememtary Tables.

## Data Availability

The whole genome sequencing data of the 1000 Genomes Project are available at https://www.internationalgenome.org/. The cancer genome sequencing data with dbGap ID phs000178.v11.p8. were downloaded from the TCGA Research Network at https://www.cancer.gov/tcga. The COSMIC 4970 indels were downloaded from COSMIC (v90) at https://cancer.sanger.ac.uk/cosmic. The GATK Resource Bundle is available at https://storage.cloud.google.com/genomics-public-data/resources/broad/hg38/v0/Mills_and_1000G_gold_standard.indels.hg38.vcf.gz

## Abbreviations

3D: 3-Dimensional
Indel: Insertion and deletion
SNP: Single nucleotide polymorphism
SV: Structural variant
NGS: Next generation sequencing
GATK_UG: GATK unified genotyper
GATK_HC: GATK Haplotypecaller
FN: False negative
TP: True positive
FP: False positive
FS: Frame-shift
NFS: Non-frame-shift
COSMIC: The catalogue of somatic mutation in cancer
TCGA: The cancer genome atlas

## References

[1] Auton A, Brooks LD, Durbin RM, Garrison EP, Kang HM, Korbel JO, Marchini JL, McCarthy S, McVean GA (2015). A global reference for human genetic variation. Nature.

[2] Baker M (2012). Structural variation: the genome's hidden architecture. Nat Methods.

[3] Cameron DL, Di Stefano L, Papenfuss AT (2019). Comprehensive evaluation and characterisation of short read general-purpose structural variant calling software. Nat Commun.

[4] O'Leary NA, Wright MW, Brister JR, Ciufo S, Haddad D, McVeigh R, Rajput B, Robbertse B, Smith-White B, Ako-Adjei D (2016). Reference sequence (RefSeq) database at NCBI: current status, taxonomic expansion, and functional annotation. Nucleic Acids Res.

[5] Stephens PJ, Tarpey PS, Davies H, Van Loo P, Greenman C, Wedge DC, Nik-Zainal S, Martin S, Varela I, Bignell GR (2012). The landscape of cancer genes and mutational processes in breast cancer. Nature.

[6] Lappalainen I, Lopez J, Skipper L, Hefferon T, Spalding JD, Garner J, Chen C, Maguire M, Corbett M, Zhou G (2013). DbVar and DGVa: public archives for genomic structural variation. Nucleic Acids Res.

[7] Sherry ST, Ward MH, Kholodov M, Baker J, Phan L, Smigielski EM, Sirotkin K (2001). dbSNP: the NCBI database of genetic variation. Nucleic Acids Res.

[8] Tate JG, Bamford S, Jubb HC, Sondka Z, Beare DM, Bindal N, Boutselakis H, Cole CG, Creatore C, Dawson E (2019). COSMIC: the catalogue of somatic mutations in cancer. Nucleic Acids Res.

[9] Mills RE, Luttig CT, Larkins CE, Beauchamp A, Tsui C, Pittard WS, Devine SE (2006). An initial map of insertion and deletion (INDEL) variation in the human genome. Genome Res.

[10] Mills RE, Pittard WS, Mullaney JM, Farooq U, Creasy TH, Mahurkar AA, Kemeza DM, Strassler DS, Ponting CP, Webber C (2011). Natural genetic variation caused by small insertions and deletions in the human genome. Genome Res.

[11] Niu B, Scott AD, Sengupta S, Bailey MH, Batra P, Ning J, Wyczalkowski MA, Liang W-W, Zhang Q, McLellan MD (2016). Protein-structure-guided discovery of functional mutations across 19 cancer types. Nat Genet.

[12] Imielinski M, Guo G, Meyerson M (2017). Insertions and deletions target lineage-defining genes in human cancers. Cell.

[13] Albers CA, Lunter G, MacArthur DG, McVean G, Ouwehand WH, Durbin R (2011). Dindel: accurate indel calls from short-read data. Genome Res.

[14] Poplin R, Ruano-Rubio V, DePristo M, Fennell T, Carneiro M, Van der Auwera G, Kling D, Gauthier L, Onder S, Levy-Moonshine A et al: Scaling accurate genetic variant discovery to tens of thousands of samples. *bioRxiv* 2018.

[15] DePristo MA, Banks E, Poplin R, Garimella KV, Maguire JR, Hartl C, Philippakis AA, del Angel G, Rivas MA, Hanna M (2011). A framework for variation discovery and genotyping using next-generation DNA sequencing data. Nat Genet.

[16] Ye K, Schulz MH, Long Q, Apweiler R, Ning Z (2009). Pindel: a pattern growth approach to detect break points of large deletions and medium sized insertions from paired-end short reads. Bioinformatics.

[17] Rimmer A, Phan H, Mathieson I, Iqbal Z, Twigg SRF, Wilkie AOM, McVean G, Lunter G (2014). Integrating mapping-, assembly- and haplotype-based approaches for calling variants in clinical sequencing applications. Nat Genet.

[18] Li H, Handsaker B, Wysoker A, Fennell T, Ruan J, Homer N, Marth G, Abecasis G, Durbin R (2009). Genome project data processing S: the sequence alignment/map format and SAMtools. Bioinformatics.

[19] Koboldt DC, Chen K, Wylie T, Larson DE, McLellan MD, Mardis ER, Weinstock GM, Wilson RK, Ding L (2009). VarScan: variant detection in massively parallel sequencing of individual and pooled samples. Bioinformatics.

[20] Cibulskis K, Lawrence MS, Carter SL, Sivachenko A, Jaffe D, Sougnez C, Gabriel S, Meyerson M, Lander ES, Getz G (2013). Sensitive detection of somatic point mutations in impure and heterogeneous cancer samples. Nat Biotechnol.

[21] Benjamin D, Sato T, Cibulskis K, Getz G, Stewart C, Lichtenstein L: Calling Somatic SNVs and Indels with Mutect2. *BioRxiv* 2019:861054.

[22] Saunders CT, Wong WS, Swamy S, Becq J, Murray LJ, Cheetham RK (2012). Strelka: accurate somatic small-variant calling from sequenced tumor-normal sample pairs. Bioinformatics.

[23] Kim S, Scheffler K, Halpern AL, Bekritsky MA, Noh E, Kallberg M, Chen X, Kim Y, Beyter D, Krusche P (2018). Strelka2: fast and accurate calling of germline and somatic variants. Nat Methods.

[24] Koboldt DC, Zhang Q, Larson DE, Shen D, McLellan MD, Lin L, Miller CA, Mardis ER, Ding L, Wilson RK (2012). VarScan 2: somatic mutation and copy number alteration discovery in cancer by exome sequencing. Genome Res.

[25] Hasan MS, Wu X, Zhang L (2015). Performance evaluation of indel calling tools using real short-read data. Hum Genomics.

[26] Carter SL, Cibulskis K, Helman E, McKenna A, Shen H, Zack T, Laird PW, Onofrio RC, Winckler W, Weir BA (2012). Absolute quantification of somatic DNA alterations in human cancer. Nat Biotechnol.

[27] Liu X, Wang J, Chen L (2013). Whole-exome sequencing reveals recurrent somatic mutation networks in cancer. Cancer Lett.

[28] Abel HJ, Duncavage EJ (2013). Detection of structural DNA variation from next generation sequencing data: a review of informatic approaches. Cancer Genet.

[29] Kroigard AB, Thomassen M, Laenkholm AV, Kruse TA, Larsen MJ (2016). Evaluation of nine somatic variant callers for detection of somatic mutations in exome and targeted deep sequencing data. PLoS ONE.

[30] Xu C (2018). A review of somatic single nucleotide variant calling algorithms for next-generation sequencing data. Comput Struct Biotechnol J.

[31] Cai L, Yuan W, Zhang Z, He L, Chou KC (2016). In-depth comparison of somatic point mutation callers based on different tumor next-generation sequencing depth data. Sci Rep.

[32] Roberts ND, Kortschak RD, Parker WT, Schreiber AW, Branford S, Scott HS, Glonek G, Adelson DL (2013). A comparative analysis of algorithms for somatic SNV detection in cancer. Bioinformatics.

[33] Spencer DH, Tyagi M, Vallania F, Bredemeyer AJ, Pfeifer JD, Mitra RD, Duncavage EJ (2014). Performance of common analysis methods for detecting low-frequency single nucleotide variants in targeted next-generation sequence data. J Mol Diagn.

[34] Wang Q, Jia P, Li F, Chen H, Ji H, Hucks D, Dahlman KB, Pao W, Zhao Z (2013). Detecting somatic point mutations in cancer genome sequencing data: a comparison of mutation callers. Genome Med.

[35] GATK Resource Bundle. https://storage.cloud.google.com/genomics-public-data/resources/broad/hg38/v0/Mills_and_1000G_gold_standard.indels.hg38.vcf.gz

[36] Zhao H, Yang Y, Lin H, Zhang X, Mort M, Cooper DN, Liu Y, Zhou Y (2013). DDIG-in: discriminating between disease-associated and neutral non-frameshifting micro-indels. Genome Biol.

[37] Folkman L, Yang Y, Li Z, Stantic B, Sattar A, Mort M, Cooper DN, Liu Y, Zhou Y (2015). DDIG-in: detecting disease-causing genetic variations due to frameshifting indels and nonsense mutations employing sequence and structural properties at nucleotide and protein levels. Bioinformatics.

[38] Pagel KA, Antaki D, Lian A, Mort M, Cooper DN, Sebat J, Iakoucheva LM, Mooney SD, Radivojac P (2019). Pathogenicity and functional impact of non-frameshifting insertion/deletion variation in the human genome. PLoS Comput Biol.

[39] Lin M, Whitmire S, Chen J, Farrel A, Shi X (2017). Guo J-t: Effects of short indels on protein structure and function in human genomes. Scientific reports.

[40] Ferlaino M, Rogers MF, Shihab HA, Mort M, Cooper DN, Gaunt TR, Campbell C (2017). An integrative approach to predicting the functional effects of small indels in non-coding regions of the human genome. BMC Bioinformatics.

[41] Krawitz P, Rodelsperger C, Jager M, Jostins L, Bauer S, Robinson PN (2010). Microindel detection in short-read sequence data. Bioinformatics.

[42] Brogna S, Wen J (2009). Nonsense-mediated mRNA decay (NMD) mechanisms. Nat Struct Mol Biol.

[43] Pleasance ED, Cheetham RK, Stephens PJ, McBride DJ, Humphray SJ, Greenman CD, Varela I, Lin M-L, Ordóñez GR, Bignell GR (2010). A comprehensive catalogue of somatic mutations from a human cancer genome. Nature.

[44] Nik-Zainal S, Alexandrov LB, Wedge DC, Van Loo P, Greenman CD, Raine K, Jones D, Hinton J, Marshall J, Stebbings LA (2012). Mutational processes molding the genomes of 21 breast cancers. Cell.

[45] Li H, Durbin R (2009). Fast and accurate short read alignment with Burrows-Wheeler transform. Bioinformatics.

[46] Sathya B, Dharshini AP, Kumar GR (2015). NGS meta data analysis for identification of SNP and INDEL patterns in human airway transcriptome: a preliminary indicator for lung cancer. Appl Transl Genom.

