# Supplementary material for: Comparative assessments of indel annotations in healthy and cancer genomes with next-generation sequencing data
Source: BMC Med Genomics. 2020 Nov 10;13:170. doi: 10.1186/s12920-020-00818-6 (PMC7653722; doi:10.1186/s12920-020-00818-6)
Supplement: Supplementary file 1 — Additional file 1. Supplememtary Tables. [file 12920_2020_818_MOESM1_ESM.docx]

**Table S1. Indel length (bps) distribution from different programs**

| **Programs** | **1-10** | **11-20** | **21-31** | **31-40** | **41-50** | **>50** |
| --- | --- | --- | --- | --- | --- | --- |
| **Varscan** | 973,285  (98.375%) | 14,468  (1.462%) | 1,338  (0.135%) | 168  (0.017%) | 52  (0.005%) | 55  (0.006%) |
| **GATK_UG** | 3,258,954  (97.005%) | 85,365  (2.541%) | 11,972  (0.356%) | 2,145  (0.064%) | 533  (0.016%) | 604  (0.018%) |
| **GATK_HC** | 3,577,313  (90.730%) | 251,263  (6.373%) | **63,270**  (1.605%) | **23,372**  (0.593%) | **10,451**  (0.265%) | 17,157  (0.435%) |
| **Pindel** | 1,015,404  (89.544%) | 56,060  (4.944%) | 19,085  (1.683%) | 9,412  (0.830%) | 7,268  (0.641%) | **26,746**  (2.359%) |
| **Dindel** | 4,975,421  (94.443%) | 244,824  (4.647%) | 36,877  (0.700%) | 6,746  (0.128%) | 1,839  (0.035%) | 2,442  (0.046%) |
| **Platypus** | 5,644,495  (96.617%) | 168,210  (2.879%) | 23,381  (0.400%) | 4,153  (0.071%) | 987  (0.017%) | 886  (0.015%) |
| **SAMTools** | **19,903,777**  (98.113%) | **326,069**  (1.607%) | 45,619  (0.225%) | 7,891  (0.039%) | 1,940  (0.010%) | 1,389  (0.007%) |
| **Benchmark** | 1,218,248  (95.944%) | 33,112  (2.608%) | 8,550  (0.673%) | 3,817  (0.301%) | 2,025  (0.159%) | 4,002  (0.315%) |

**Table S2. Indel types (deletion and insertion)**

| **Tool** | **Deletion*** | **TP Deletion^#^** | **Insertion*** | **TP Insertion^#^** |
| --- | --- | --- | --- | --- |
| **Varscan** | 532,457  (55.59%) | 302,541  (41.23%) | 425,384  (44.41%) | 230,560  (42.63%) |
| **GATK_UG** | 1,330,229  (49.50%) | 491,384  (66.97%) | 1,357,011  (50.50%) | 393,379  (72.74%) |
| **GATK_HC** | 1,512,471  (50.83%) | 544,906  (74.26%) | 1,463,170  (49.17%) | 403,832  (74.67%) |
| **Pindel** | 594,809  (55.77%) | 246,059  (33.53%) | 471,659  (44.23%) | 200,563  (37.08%) |
| **Dindel** | 2,299,063  (56.18%) | **572,579**  **(78.03%)** | 1,793,001  (43.82%) | **422,368**  **(78.10%)** |
| **Platypus** | 2,351,612  (54.13%) | 548,043  (74.69%) | 1,992,999  (45.87%) | 393,003  (72.67%) |
| **SAMTools** | 10,467,240  **(65.36%)** | 550,396  (75.01%) | 5,547,278  (34.64%) | 380,464  (70.35%) |
| **Benchmark** | 733,758  (57.57%) | - | 540,822  (42.43%) | - |
| *****:Percentage = percentage of deletions/insertions of indels by each program  **^#^**:Percentage = percentage of deletions/insertions in benchmark by each program | | | | |

**Table S3. Coding indel types (NFS and FS)**

| **Tools** | **FS*** | **TP FS^#^** | **NFS*** | **TP NFS^#^** |
| --- | --- | --- | --- | --- |
| **Varscan** | 645  (57.95%) | 401  (37.79%) | 468  (42.05%) | 263  (33.04%) |
| **GATK_UG** | 1,460  (57.46%) | 620  (58.44%) | 1,081  (42.54%) | 491  (61.68%) |
| **GATK_HC** | 2,826  (60.57%) | 703  (66.26%) | 1,840  (39.43%) | 550  (69.10%) |
| **Pindel** | 1,099  (57.09%) | 363  (34.21%) | 826  (42.91%) | 271  (34.05%) |
| **Dindel** | 10,745  (83.11%) | 743  (70.03%) | 2,183  (16.89%) | 579  (72.74%) |
| **Platypus** | 4,550  (69.96%) | 722  (68.05%) | 1,954  (30.04%) | 596  (74.87%) |
| **SAMTools** | **113,244**  **(94.88%)** | **790**  **(74.46%)** | **6,111**  (5.12%) | **610**  **(76.63%)** |
| **Benchmark** | 1,061  (57.14%) | - | 796  (42.86%) | - |
| *****:Percentage = percentage of FS or NFS of indels by each program  **^#^**:Percentage = percentage of FS or NFS in benchmark by each program | | | | |

**Table S4. Distribution of somatic indel lengths**

| **Tools / Cancer types** | **1-10** | **11-20** | **21-31** | **31-40** | **41-50** | **>50** |
| --- | --- | --- | --- | --- | --- | --- |
| **Strelka2** | 12,699  (97.93%) | 219  (1.69%) | 43  (0.33%) | 6  (0.05%) | 0  (0.00%) | 0  (0.00%) |
| **Strelka** | 21,370  (97.41%) | 471  (2.15%) | 84  (0.38%) | 12  (0.05%) | 2  (0.01%) | 0  (0.00%) |
| **Mutect2** | **80,177**  (84.54%) | **9,514**  (10.03%) | **3,295**  (3.47%) | **1,280**  (1.35%) | **368**  (0.39%) | **202**  (0.21%) |
| **Varscan2** | 11,505  (96.79%) | 313  (2.63%) | 63  (0.53%) | 5  (0.04%) | 0  (0.00%) | 0  (0.00%) |
| **Bladder** | 20,629  (86.00%) | 2,527  (10.53%) | 695  (2.90%) | 91  (0.38%) | 15  (0.06%) | 31  (0.13%) |
| **Breast** | 48,136  (84.83%) | 5,692  (10.03%) | 1,823  (3.21%) | 801  (1.41%) | 206  (0.36%) | 89  (0.16%) |
| **Colon** | 56,986  (93.58%) | 2,298  (3.77%) | 967  (1.59%) | 411  (0.67%) | 149  (0.24%) | 82  (0.13%) |
| **Germline** | 1,215,718  (95.95%) | 33,004  (2.60%) | 8,505  (0.67%) | 3,795  (0.30%) | 2,012  (0.16%) | 3,974  (0.31%) |

**Table S5. Somatic indel types (deletion and insertion)**

| **Cancer Types** | **Tools** | **# of Deletions** | **Deletion Percentage** | **#of Insertions** | **Insertion Percentage** |
| --- | --- | --- | --- | --- | --- |
| **Bladder** | **Strelka** | 1,417 | 64.73% | 772 | 35.27% |
|  | **Strelka2** | 614 | 70.49% | 257 | 29.51% |
|  | **Varscan2** | 1,128 | 62.53% | 676 | 37.47% |
|  | **Mutect2** | 7,784 | 40.03% | 11,662 | 59.97% |
| **Breast** | **Strelka** | 3,186 | 57.57% | 2,348 | 42.43% |
|  | **Strelka2** | 1,413 | 65.24% | 753 | 34.76% |
|  | **Varscan2** | 2,227 | 58.67% | 1,569 | 41.33% |
|  | **Mutect2** | 16,273 | 35.40% | 29,701 | 64.60% |
| **Colon** | **Strelka** | 9,280 | 65.28% | 4,936 | 34.72% |
|  | **Strelka2** | 6,973 | 70.22% | 2,957 | 29.78% |
|  | **Varscan2** | 5,058 | 80.46% | 1,228 | 19.54% |
|  | **Mutect2** | 20,022 | 63.74% | 11,390 | 36.26% |
| **Germline indels** | | 731,665 | 57.75% | 535,343 | 42.25% |
| **COSMIC indels** | | 3,104 | 52.00% | 1,709 | 34.39% |

**Table S6. Somatic coding indel types (FS and NFS)**

| **Cancer Types** | **Tools** | **# of FS indels** | **FS Percentage** | **# of NFS indels** | **NFS Percentage** |
| --- | --- | --- | --- | --- | --- |
| **Bladder** | **Strelka** | 135 | 53.78% | 116 | 46.22% |
|  | **Strelka2** | 6 | 20.69% | 23 | 79.31% |
|  | **Varscan2** | 66 | 63.46% | 38 | 36.54% |
|  | **Mutect2** | 4,292 | 63.25% | 2,494 | 36.75% |
| **Breast** | **Strelka** | 142 | 62.28% | 86 | 37.72% |
|  | **Strelka2** | 4 | 80.00% | 1 | 20.00% |
|  | **Varscan2** | 60 | 71.11% | 30 | 28.89% |
|  | **Mutect2** | 7,651 | 71.11% | 3,109 | 28.89% |
| **Colon** | **Strelka** | 303 | 78.29% | 84 | 21.71% |
|  | **Strelka2** | 37 | 88.10% | 5 | 11.90% |
|  | **Varscan2** | 202 | 82.79% | 42 | 17.21% |
|  | **Mutect2** | 3,218 | 65.17% | 1,720 | 34.83% |
| **Germline indels** | | 697 | 48.37% | 744 | 51.63% |
| **COSMIC indels** | | 2,515 | 81.05% | 588 | 18.95% |
